# Coordinate attention and deformable convolution-enhanced YOLO for bilateral maxillary sinus segmentation, a paranasal sinus structure, in CT images

**DOI:** 10.3389/fmed.2026.1945669

**Published:** 2026-09-25

**Authors:** Ameera Jaradat, Salem Alhatamleh, Mohammad Amin, Abrar Omari, Amal Alishwait, Rabia Emhamed Al Mamlook, Abdullah Mohammed Al Fatais

**Affiliations:** 1 Computer Science Department, Faculty of Information Technology and Computer Sciences, Yarmouk University, Irbid, Jordan; 2 Computer Science & Engineering, College of Engineering, American University of Sharjah, Sharjah, United Arab Emirates; 3 Prince Hamzah Hospital, Amman, Jordan; 4 Department of Business Administration, Trine University, Angola, IN, United States; 5 Department of Artificial Intelligence, Center of Advanced Research for Complementary Medicine, University of Zawia, Zawia, Libya; 6 Department of Industrial Engineering, College of Engineering, King Khalid University, Abha, Saudi Arabia; 7 Center for Engineering and Technology Innovations, King Khalid University, Abha, Saudi Arabia

**Keywords:** computed tomography, coordinate attention, deep learning, deformable convolution, instance segmentation, maxillary sinus segmentation, paranasal sinus, YOLO

## Abstract

Accurate delineation of the maxillary sinuses in CT images is a relevant step supporting preoperative planning and the evaluation of sinonasal disease within otolaryngology. Manual contouring of these paranasal sinus structures is difficult and error-prone, since the surrounding anatomy is complex, bony structures are thin, and inter-patient variability is large. This paper proposes an instance segmentation framework that extends the YOLO11n-Seg architecture through the integration of Coordinate Attention (CoordAtt) modules and Deformable Convolutional Networks v2 (DCNv2), to improve spatial feature representation and boundary precision for the right and left maxillary sinuses in CT images. CoordAtt encodes directional spatial context along horizontal and vertical axes to enhance anatomical localisation, while DCNv2 replaces selected fixed-kernel convolution layers with adaptive deformable sampling to better capture irregular and thin-walled sinus boundaries. The proposed model was trained and evaluated on the publicly available NasalSeg dataset, using all axial CT slices containing either maxillary sinus from 130 patients (3,620 slices; 104/13/13 patients across training/validation/test), and compared against three YOLO-family baselines (YOLOv8n-Seg, YOLO11n-Seg, and YOLO26n-Seg). On the 382-slice test set (13 patients), the proposed model achieved a mask mAP50 of 93.07% and mask mAP50-95 of 74.49%, outperforming the three baselines by up to +4.60 and +3.30 percentage points, respectively, and a box mAP50 of 91.00% and mAP50-95 of 75.83%. A component-wise ablation study confirmed that this improvement arises from the joint use of CoordAtt and DCNv2 rather than from either mechanism alone, and patient-level bootstrap confidence intervals and paired significance testing confirmed the statistical robustness of the observed gains. These results represent proof-of-concept evidence that coordinate-aware attention and deformable convolution can be effectively integrated into a YOLO-based instance segmentation pipeline for bilateral maxillary sinus delineation from CT.

## 1 Introduction

The nasal cavity serves as an important conditioning channel for inspired air through its respective filtration/humidification/temperature regulation functions, in addition to contributing to olfaction and the mucosal immune response. Due to its various anatomical layout and close anatomic relationships with the paranasal sinuses, orbit and anterior cranial fossa this region is vulnerable to many diseases including chronic rhinosinusitis (CRS), nasal polyps, septal deviation and sinonasal neoplasms. These disorders compromise normal nasal physiology, impair quality of life and can cause severe local complications (1).

These disorders include chronic rhinosinusitis, which is the most prevalent inflammatory disease of the upper respiratory tract, with an estimated global prevalence ranging between 8% and 10.9%, as stated by various epidemiological studies (2). This disease carries a lot of health implications both clinically and socially, as well as economically due to its persistence in symptoms, functional impairments and increased utilization of healthcare resources. In this regard, there must be knowledge concerning the anatomy of sinonasal region coupled with accurate diagnosis and preoperative planning to make sure that patients get favorable results (3).

The pre-operative workup for FESS primarily relies on imaging modalities to determine the extent of sinonasal pathology and the anatomic characteristics of the patients (1). High-resolution CT scans are generally done on routine basis since they provide clear visualization of the paranasal sinuses and surrounding osseous structures. The anatomic information gained through CT scans enables the surgeons to have a clear idea about the unique anatomy of the sinonasal region of the patients. Anatomical variations and important surgical landmarks can be detected beforehand (4). MRI is especially helpful in situations where CT is unable to completely describe the nature of the lesion in the sinonasal cavity. It gives better soft-tissue contrast which enables differentiation of inflammatory tissue, retained secretions, and tumors. Combined evaluation of MRI and CT helps in determining the disease status, anatomical variation, and making appropriate decisions prior to surgery (5).

Nevertheless, accurate segmentation of the maxillary sinuses remains a demanding task. Although manual segmentation continues to be considered the reference standard, it requires substantial time and expert input and is susceptible to variability between observers. Delineating the interfaces between air, bone, and soft tissue is often difficult, particularly in patients with distorted anatomy caused by chronic inflammatory disease or previous surgical intervention. Such factors may reduce the consistency and reproducibility of segmentation results in both clinical practice and research. Consequently, automated segmentation methods have attracted increasing attention as a means of reducing observer dependency, improving workflow efficiency, and promoting more standardized analysis of sinonasal imaging data (6, 7).

Even with all the research in this field, there are still certain deficiencies in current methods that necessitate the study proposed herein. The state-of-the-art volumetric segmenters currently used, whether ensemble models like 3D-NASE (8) or frequency-domain networks like AFS-DSN (9), use 3D CT volumes and are computationally expensive, which makes them unsuitable for limited clinical settings. Lightweight YOLO-family networks have proven to perform well in real-time medical imaging applications but have not been explored for instance segmentation of the maxillary sinuses. Furthermore, standard YOLO backbones rely on fixed rectangular convolutional kernels that are poorly suited to the thin-walled, non-convex, and anatomically variable boundaries of the maxillary sinuses. No prior work has specifically investigated the integration of coordinate-aware spatial attention and deformable convolution mechanisms within a YOLO-based segmentation framework for sinonasal CT analysis.

In order to tackle these issues, this paper presents a new instance segmentation algorithm for automatic identification of the bilateral maxillary sinuses from CT scans. In this work, we enhance the light-weight YOLO11n-Seg by incorporating two new architectural elements which are: CoordAtt modules for modeling directional spatial information to assist in localization and DCNv2 for flexible sampling of features based on the irregular shape of sinus boundary. The new model has been validated on publicly available NasalSeg dataset (10), containing 130 CT volumes, where our approach is compared to three competing approaches from the YOLO family, YOLOv8n-Seg, YOLO11n-Seg, and YOLO26n-Seg.

The main contributions of this work are summarised as follows:

- A moderate-capacity instance segmentation architecture is proposed for bilateral maxillary sinus delineation from CT images, extending YOLO11n-Seg with Coordinate Attention and Deformable Convolutional Networks to improve boundary precision, at an increased but explicitly quantified computational cost relative to the lightweight YOLO11n-Seg baseline (Section 4.5).
- The proposed model achieves a mask mAP50 of 93.07% and mask mAP50-95 of 74.49% on the 382-slice NasalSeg test set (13 patients), outperforming three competitive YOLO-family baselines by up to +4.60 and +3.30 pp, respectively.
- Systematic comparison of four instance segmentation architectures of the YOLO family YOLOv8n-Seg, YOLO11n-Seg, YOLO26n-Seg, and the proposed model is performed under the same training setup to enable a controlled, internal comparison under identical training conditions for maxillary sinus CT segmentation.

The remainder of this paper is organised as follows. Section 2 reviews related work on deep learning-based sinonasal segmentation and YOLO-family architectures. Section 3 describes the dataset, preprocessing pipeline, and proposed model architecture. Section 4 presents the experimental results and quantitative comparisons. Section 5 discusses the findings and limitations of the proposed approach. Section 6 concludes the paper and outlines directions for future work.

## 2 Related work

### 2.1 Nasal cavity and paranasal sinus segmentation

NasalSeg is the name of Zhang et al. (10) dataset, which has been designed as the first large-scale publicly available dataset for automatic segmentation of the nasal cavity and paranasal sinuses from 3D CT volumes. This dataset contains 130 scans and each one has voxel-wise annotation of five anatomical structures that have been annotated by three experienced annotators and validated by otolaryngologists and radiologists who have 20 years of professional experience. The authors have also set up an nnU-Net baseline with five-fold cross validation with a mean Dice score of 58.55% ± 5.43%.

Building directly upon this benchmark, Pani et al. (8) proposed 3D-NASE, an ensemble framework that integrates six complementary volumetric architectures 3D U-Net, UNETR, Swin UNETR, SegResNet, DAF3D, and V-Net through majority and soft voting strategies on the same NasalSeg dataset. The soft voting ensemble obtained an average Dice score of 94.43%, demonstrating a 35.88% performance enhancement compared to the nnU-Net baseline and a 4.52% decrease in inter-fold variability. Even though there was a remarkable enhancement in the system performance, the method needs six models for simultaneous predictions, thereby resulting in high computation cost.

The researchers Wan and Chen (9) designed a novel model known as Adaptive Frequency-Spatial Dual-Stream Network (AFS-DSN). It is designed to create complementary spatial and frequency domain representations for the task of 3D nasal cavity and paranasal sinus segmentation. This model combines the advantages of the spatial U-net like model with those of the frequency branch which uses the 3D Discrete Wavelet Transformation on three different Daubechies wavelets (db1, db2, db4) resulting in 24 frequency sub-bands which pass through learnable band-wise weighting. Evaluated on NasalSeg with a 70/15/15 train/validation/test split, AFS-DSN achieved an overall Dice score of 94.34% ± 2.30% on the independent test set, with statistically significant improvements in thin-wall regions (91.34% vs. 90.57% baseline, *p* = 0.004) and Surface Dice at 1 mm tolerance (0.874 vs. 0.868 baseline, *p* = 0.010). The authors further introduced AFS-DSN-Lite, a parameter-efficient variant (27.41M parameters) achieving comparable performance (94.37% Dice) through depthwise separable convolutions.

Alam et al. (11) proposed UHCT-Net, a unified hybrid CNN-Transformer framework for joint 3D nasal cavity segmentation and tumour analysis from CT images. The architecture employs two parallel pathways a CNN pathway for local feature extraction and a Transformer pathway for global contextual modeling fused through a learnable gated module that adaptively weights local and global representations. Anatomy-aware spatial attention (AASA) directs the network toward medically relevant anatomical regions, while multi-scale contrastive learning (MSCL) sharpens tissue boundaries in radiologically ambiguous areas. A tumor classification model is used to predict the chance of malignancy using the technique Grad-CAM for explaining activations. The network was tested on the NasalSeg dataset where the Dice score, mIoU, and overall accuracy obtained by the network were 95.08%, 90.15%, and 99.79%, respectively, performing significantly better than 3D-NASE (Dice score of 94.43%) and nnU-Net baseline (*p* < 0.012 for nnU-Net; *p* < 0.03).

### 2.2 MRI-Based sinonasal segmentation and clinical scoring

PARASIDE (12) by Möller et al. is the first automated software system for whole-paranasal sinus segmentation on T1-weighted MRI scans to evaluate the severity of chronic rhinosinusitis (CRS). The nnU-Net was used to train the model on 292 subjects of the SHIP population cohort, employing an iterative annotation-training protocol for segmentation of air and soft-tissue volumes of 16 annotated structures of paranasal sinuses. The segmentation model achieved an overall Dice of 0.882 ± 0.138 and an average symmetric surface distance of 0.311 ± 0.354 mm, with air volumes (Dice = 0.956 ± 0.063) consistently outperforming soft tissue segmentation (Dice = 0.796 ± 0.150). Quantitative features derived from the predicted segmentations including soft tissue volume ratios, surface coverage, and intensity averages predicted the Total Lund-Mackay Score with an MSE of 2.444 and MAE of 1.181, significantly outperforming existing volumetric scoring baselines (MSE = 3.784, MAE = 1.445), demonstrating that MRI-based automated segmentation can support objective CRS severity quantification without ionising radiation.

Ullah et al. (13) introduced an explainable hybrid CNN-Transformer framework incorporating self-supervised learning for structural analysis of paranasal sinus CT volumes. The framework combines a Swin-UNETR-based segmentation backbone with a 3D convolutional autoencoder trained on normal sinus regions to generate reconstruction residual maps highlighting structural deviations from standard osseous patterns. A 3D CNN feature extractor processes the concatenation of the CT volume, segmentation mask, and residual map to generate morphology-aware structural embeddings for downstream clinical analysis. Evaluated on the multi-institutional CT-SCOPE dataset, the hybrid segmentation model achieved mean Dice coefficients above 0.83 across all four sinus compartments, with maxillary sinuses yielding the highest accuracy (Dice = 0.87) and frontal sinuses the lowest (Dice = 0.81). Grad-CAM-based interpretability analysis confirmed that model attention localised consistently to clinically meaningful anatomical regions including sinus walls and ethmoidal partitions.

### 2.3 Osseous structure segmentation for surgical navigation

Sun et al. (14) addressed the specific challenge of segmenting the osseous structures of the paranasal sinuses (OSPS) for robot-assisted endoscopic and skull-base surgery, introducing an open-source dataset of CT scans and a UNet SwinTR model trained using semi-supervised learning strategies. The initial model was trained on nine *ex vivo* full head CT scans and subsequently improved through progressive semi-supervised learning incorporating pseudo-labels from 27 additional patients across diverse scanners and hospitals. On an external validation dataset recorded under different acquisition conditions, the best model (E5, incorporating data augmentation, pseudo-labelling, and post-processing) achieved a Dice score of 98.25 ± 0.9 and Hausdorff distance of 9.42 ± 5.60 mm, representing a statistically significant improvement over the fully supervised baseline (*p* = 0.0286). The authors demonstrated that semi-supervised pseudo-labelling strategies substantially improved the generalisation of osseous segmentation models across scanner manufacturers and imaging protocols, outperforming general-purpose foundation models such as SAM and MedSAM on this specialised anatomical target.

### 2.4 Maxillary sinus analysis and surgical planning

Hwang et al. (15) proposed SinusC-Net, a two-stage deep learning framework for automatically classifying surgical approaches for maxillary sinus floor augmentation in implant planning from CBCT images. The detection stage employed a cascaded volumetric regression network incorporating convolutional LSTM, multi-scale inputs, and deep supervision to localise five anatomical landmarks, achieving a mean radial error of 0.87 mm and successful detection rate of 95.47% at a 2 mm threshold. The subsequent 3D distance-guided classification network used predicted landmark positions as geometric priors to classify CBCT volumes into five surgical approach categories, achieving overall accuracy of 0.97, sensitivity of 0.92, specificity of 0.98, and AUC of 0.95 on 53 test volumes from three scanner systems. Ablation analysis confirmed that both the distance priors and the convLSTM, multi-scale input, and deep supervision modules contributed independently to classification performance.

More directly relevant to the present study, Altun et al. (16) developed a two-stage deep learning pipeline for automatic maxillary sinus segmentation and pathology classification on cone-beam CT images, first segmenting the maxillary sinus region and subsequently classifying the segmented region for the presence of sinus pathology. While this work targets the same anatomical structure investigated in the present study, it differs in dimensionality (3-D CBCT volumetric segmentation vs. the 2-D, slice-wise instance segmentation approach adopted here), in modality (CBCT vs. conventional CT), and in scope (a segmentation-then-classification pipeline aimed at pathology detection, rather than instance segmentation as an end in itself). This distinction motivates the present work's focus on a lightweight, single-stage instance-segmentation architecture suitable for rapid, purely anatomical delineation of the maxillary sinuses, which could subsequently feed into a downstream classification stage such as that proposed by Altun et al., rather than performing pathology classification directly.

### 2.5 YOLO-based and attention-/deformable-enhanced medical image segmentation

Beyond volumetric sinonasal segmentation, several lines of work are directly relevant to the architectural design adopted in this study. Coordinate Attention has been incorporated into medical image segmentation architectures in several contexts: Ding et al. (17) proposed CAT-Unet, enhancing a U-Net backbone with coordinate attention and a skip-neighbourhood attention transformer for general medical image segmentation; Chen et al. (18) combined multiscale input with coordinate attention in a transformer-augmented U-Net; and Xu and Yuan (19) applied coordinate attention to convolutional networks for automatic detection of pulmonary tuberculosis on chest x-rays, demonstrating the mechanism's utility for directional, position-aware feature encoding across diverse imaging modalities. Deformable convolution has similarly been applied in medical image segmentation and enhancement: Zhang et al. (20) proposed DA-FRNet, a continuity-focused deformable attention network for retinal vessel segmentation; Yi (21) combined deformable and standard convolutions for fine retinal vessel segmentation; and Jian et al. (22) used deformable convolution together with self-attention in a generative adversarial network for megavoltage CT enhancement in cervical cancer tomotherapy. Separately, the YOLO family of single-stage detectors has been increasingly adapted for medical image analysis, as surveyed by Ali and Zhang (23) and evaluated for medical segmentation tasks by Alanazi (24); however, to our knowledge, no prior work has combined coordinate attention and deformable convolution within a YOLO-based instance-segmentation framework specifically for sinonasal or maxillary sinus CT analysis, which is the gap addressed by the present study.

### 2.6 Computer-aided diagnosis for upper airway pathology

Bu et al. (25) have created a computer-assisted decision-making algorithm for differential diagnosis of sore throat disorders with the help of the Dynamic Uncertain Causality Graph (DUCG) approach, incorporating clinical experience of ear, nose and throat doctors in a probabilistic network model that can distinguish between 27 throat disorders. Although this work does not involve image segmentation and has limited direct architectural relevance to the present study, it illustrates the broader landscape of computer-aided diagnostic reasoning tools in otolaryngology.

## 3 Methodology

This section describes the approach that was used to design the automatic bilateral maxillary sinus segmentation. To develop such a strategy, the NasalSeg dataset was used. The dataset contains head CT images with manual voxel-wise annotations of the nasal cavity and paranasal sinus structures, of which the right and left maxillary sinuses are the focus of this study. As shown in Figure 1, firstly, the dataset needed to be preprocessed and prepared for further usage. Secondly, the proposed segmentation model is introduced, where the lightweight YOLO11n-Seg architecture is selected as the baseline network and modified through the integration of Coordinate Attention (CoordAtt) and Deformable Convolutional Networks (DCNv2) to improve spatial feature representation and segmentation performance. The NasalSeg dataset and YOLO11n-Seg architecture together form the foundation of the proposed maxillary sinus segmentation pipeline. The evaluation metrics of the proposed algorithm are then reported.

**Figure 1:**
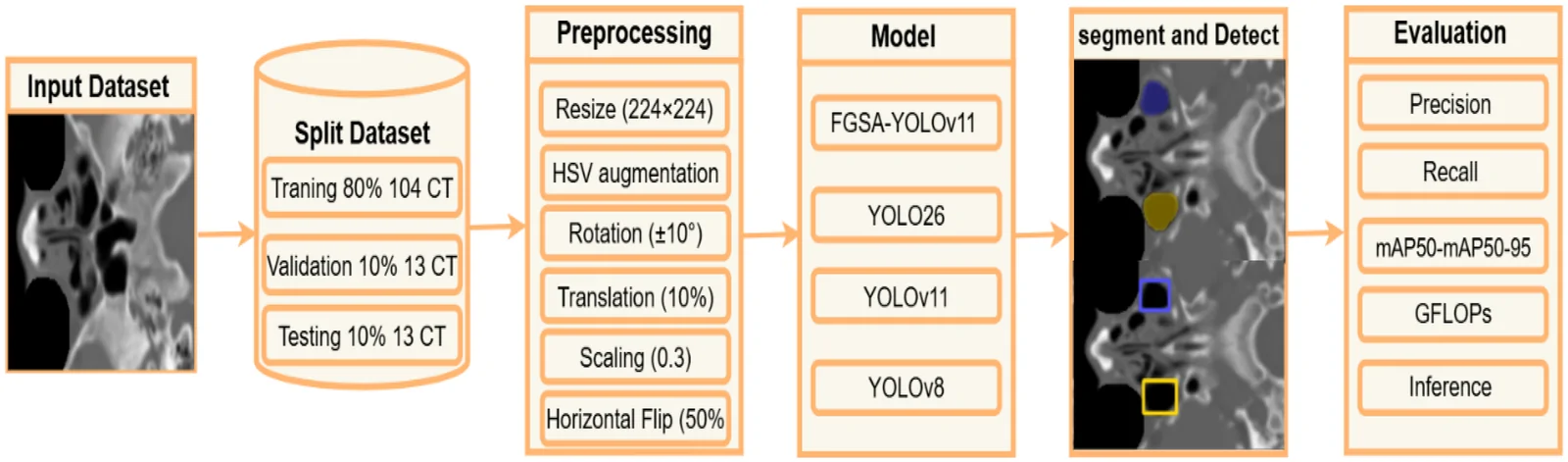
The proposed pipeline for automated maxillary sinus segmentation, comprising dataset splitting, preprocessing, model architecture comparison, segmentation output, and quantitative evaluation.

### 3.1 Dataset description and data pre-processing

The original NasalSeg dataset (10) consists of CT scans. The dataset was retrospectively collected in accordance with the relevant ethical regulations, under approval of the Institutional Review Board of Huashan Hospital, Fudan University (No. KY2024-1345); all patient-identifying information was anonymized by the original data providers using 3D Slicer, and informed consent was waived by the ethics committee given the retrospective and fully anonymized nature of data collection. The present study constitutes a secondary analysis of this publicly available, de-identified dataset; no additional patient data were collected, and no separate ethical approval was required. Collected from 130 independent patients. Each patient is represented by one three-dimensional (3D) CT volume. Each 3D volume contains multiple two-dimensional (2D) axial slices, together with a corresponding 3D segmentation mask that provides the anatomical label for each pixel or voxel. The original dataset includes six label classes: Background (Class 0), Maxillary Sinus (R) (Class 1), Maxillary Sinus (L) (Class 2), Nasal Cavity (R) (Class 3), Nasal Cavity (L) (Class 4), and Nasal Pharynx (Class 5). In this study, the segmentation task focused only on the right and left maxillary sinuses. Therefore, Class 1 and Class 2 were selected as the target classes, while Class 0 was retained as the background. Classes 3, 4, and 5, corresponding to the right nasal cavity, left nasal cavity, and nasal pharynx, respectively, were excluded and were not considered in the final segmentation task.

The dataset was divided at the patient level rather than at the individual image level. This means that all 2D slices extracted from a single patient's 3D CT scan were assigned to the same subset. Therefore, images from the same patient could not appear in more than one of the training, validation, or test sets, reducing the risk of data leakage between the different subsets. The final dataset distribution is presented in the Table 1 below.

**Table 1:** Dataset split distribution by patient and image.

Dataset split	Number of patients	Number of 2D images
Training	104	2,883
Validation	13	355
Testing	13	382
Total	130	3,620

### 3.2 Proposed model

The proposed segmentation model has been designed by modifying the lightweight YOLO11n-Seg framework through the inclusion of CoordAtt and DCNv2 blocks. YOLO11n-Seg was selected as the baseline model after consideration of the tradeoff between performance and speed. Modifications were made to the backbone to improve the extraction of features relevant to maxillary sinus delineation from head CT scans, while the multi-scale feature fusion neck and the three-scale segmentation head of the original model remain entirely unmodified.

In the modified backbone, CoordAtt modules were inserted after selected feature extraction stages to improve the representation of spatial information, whereas selected conventional convolution layers were replaced with DCNv2 layers to provide adaptive feature sampling for improved anatomical boundary representation. No additional detection or segmentation head was introduced at any stage; the extracted feature maps are processed through the original, unmodified YOLO11n-Seg feature fusion pathway and segmentation head, producing the final segmentation masks of the left and right maxillary sinuses at the same three output scales as the original architecture. The complete architecture of the proposed network, including the exact location of every integrated module, is illustrated in Figure 2, and the details of each introduced component are described in the following subsections.

**Figure 2:**
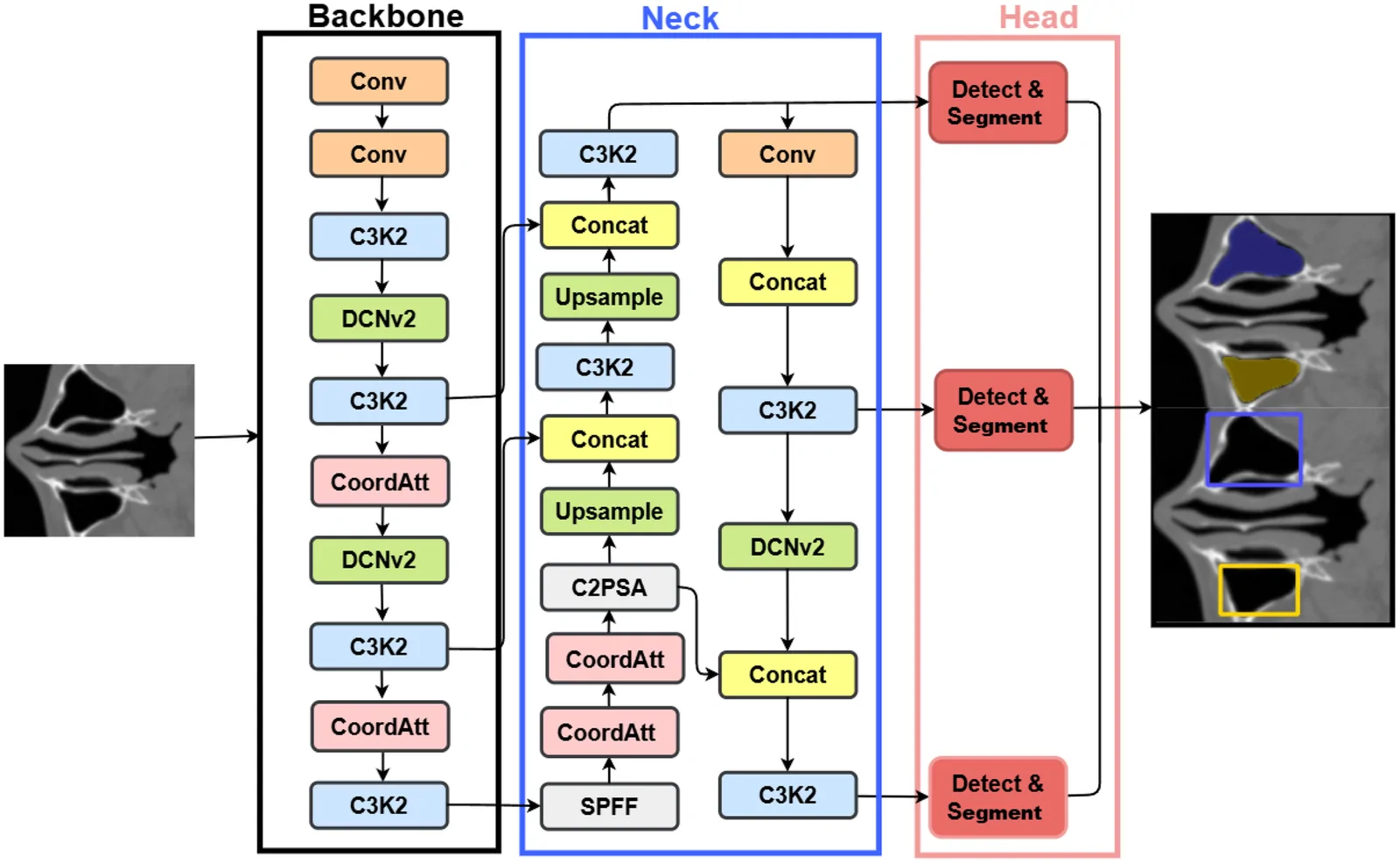
Detailed architecture of the proposed model, illustrating the modified YOLO11n-Seg backbone with DCNv2 and CoordAtt insertions, the multi-scale neck with C2PSA and feature concatenation, and the three-scale Detect & Segment head producing the final maxillary sinus segmentation masks.

#### 3.2.1 Baseline models and training configuration

The proposed model is built on the lightweight YOLO11n-Seg model architecture in the Figure 3. As the proposed framework makes changes to the network architecture alone via the introduction of the CoordAtt and DCNv2 modules without altering the training process of the baseline models, all experimental settings remain the same for both the proposed model and baseline models. This approach guarantees that the performance comparison is an accurate reflection of the impact of the architectural changes in the proposed model and not due to the variations in the training process. To evaluate the proposed model, the comparison experiments were run against the baseline models, which include YOLOv8n-Seg, YOLO11n-Seg, and YOLO26n-Seg, using the same dataset split and training hyperparameters.

**Figure 3:**
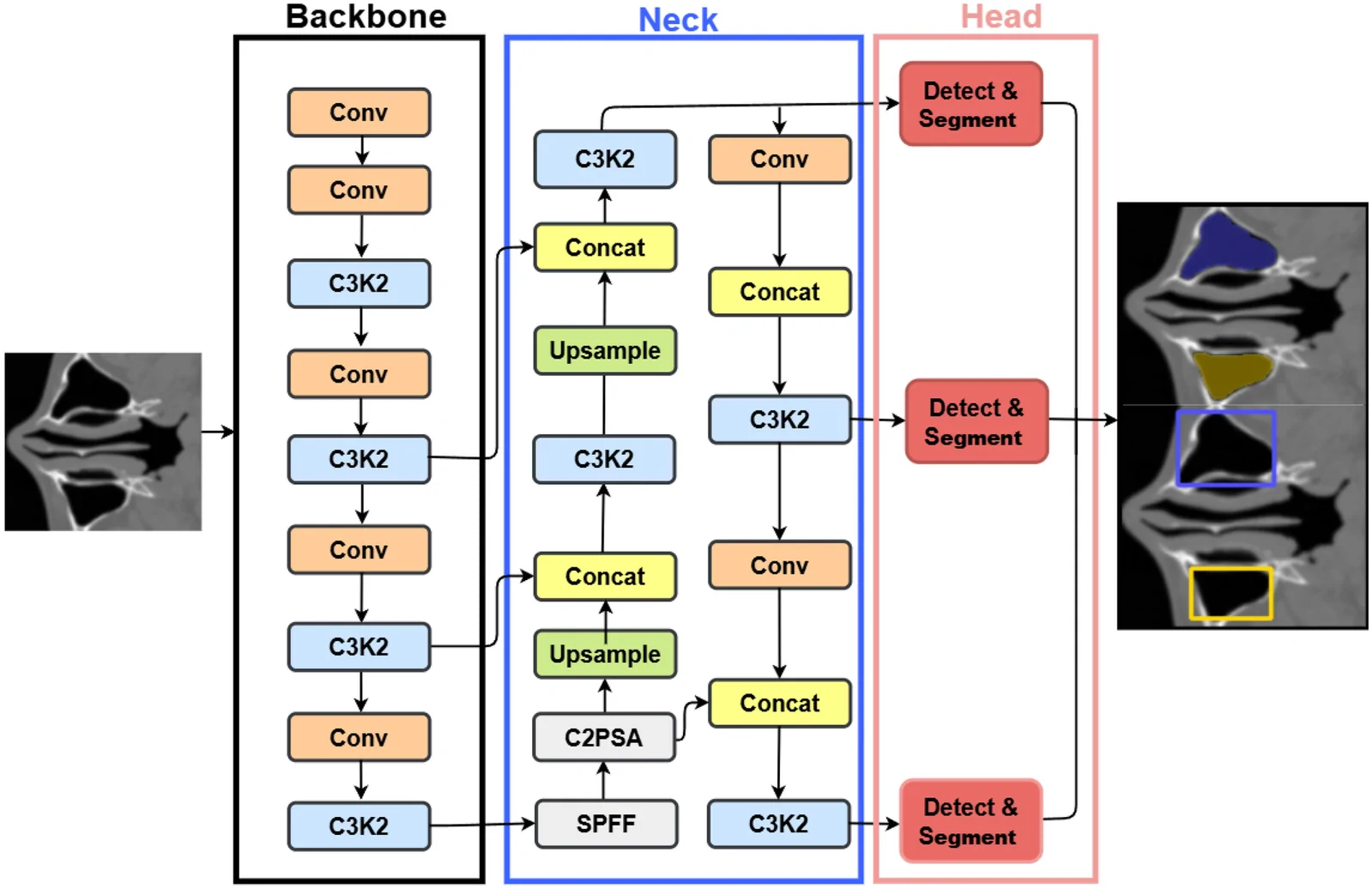
Standard YOLO11n-Seg baseline architecture consisting of the Conv/C3K2 backbone, the C2PSA/SPFF-based multi-scale neck, and the three-scale Detect & Segment head, prior to the integration of CoordAtt and DCNv2 modules.

#### 3.2.2 Coordinate attention module

The CoordAtt block was adopted for the proposed network to enhance the localisation and feature representation of the maxillary sinuses in head CT scans (17). Different from the traditional channel attention approach, which focuses only on the recalculation of the features without considering the spatial positions, CoordAtt is capable of considering the spatial position information by separating spatial coding into two directional dimensions.

This enables the network to capture long-range dependencies while preserving spatial details. Given an input feature map $X\in R^{C\times H\times W}$, the CoordAtt module performs separate global pooling operations along the horizontal and vertical directions to generate direction-aware feature representations. The formulation for the coordinate system along the horizontal and vertical axes is described in Equations 1, 2, whereby *X* is the input feature map; *C* is the number of feature maps; and *H* and *W* stand for the height and width of the feature map, respectively. The variables *c*, *h*, and *w* indicate the channel index and spatial coordinates, following the standard coordinate attention formulation adopted in prior medical imaging applications (18). The terms $z_{h}$ and $z_{w}\;$ represent the horizontal and vertical directional feature representations obtained through the pooling operations respectively. These directional representations preserve positional information and are used to generate attention weights for adaptive feature recalibration.

$$z_{h}(c,h)=\frac{1}{W}\overset{W}{\underset{i=1}{\sum }}x(c,h,i)$$ (1)

$$z_{w}(c,w)=\frac{1}{H}\overset{H}{\underset{\;j=1}{\sum }}x(c,j,w)$$ (2)

The resulting attention maps facilitate the network to focus on the relevant anatomical areas while decreasing irrelevant activations. It becomes useful during maxillary sinus segmentation, where the target areas are located within complex anatomical zones having similar intensity features on CT images. Spatial information helps in enhancing localization and extracting boundary features of the left and right maxillary sinuses (19). In the developed methodology, CoordAtt blocks were added to some of the backbone stages of YOLO11n-Seg model prior to multi-scale feature aggregation. Thus, feature representation of maxillary sinuses is enhanced without increasing computational complexity. The design of the included Coordinate Attention block is shown in Figure 4.

**Figure 4:**
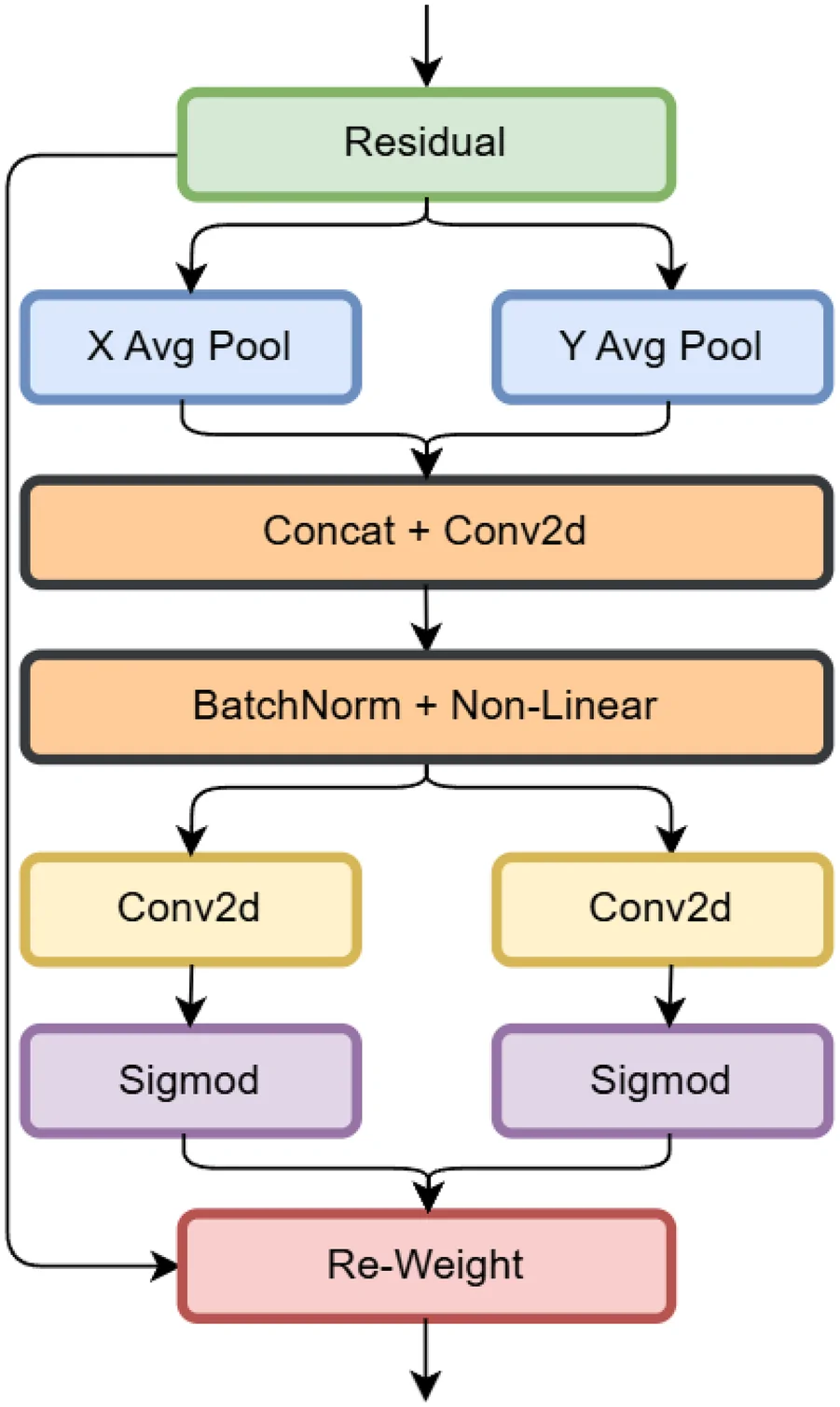
Architecture of the coordinate attention (CoordAtt) module, comprising horizontal and vertical average pooling branches, joint feature transformation via Conv2d and BatchNorm, and dual sigmoid-gated attention weight generation for directional spatial feature re-weighting.

#### 3.2.3 Deformable convolutional network v2 (DCNv2)

Incorporation of the Deformable Convolutional Network version 2 (DCNv2) module into the model became necessary to detect the complex boundaries of the maxillary sinus through the CT scan images (20). The standard convolutions use fixed sampling locations for each pixel in an image. This could be a drawback since the convolutions cannot change their sampling locations according to the shape of the region. This could cause a decrease in accuracy if the target boundaries show variability.

DCNv2 improves the flexibility of convolution by introducing learnable offsets and modulation masks, allowing the sampling locations and feature contributions to be dynamically adjusted according to the input image characteristics, consistent with its application in related medical segmentation tasks (21). Given an input feature map *X*, the offset field and modulation mask are generated using an additional convolution layer in Equations 3, 4, where ΔP represents the learned spatial offsets, *M* denotes the modulation mask, $Conv_{offset}$ and $Conv_{mask}$ indicate the convolution operations used to predict offsets and masks, respectively, and σ represents the sigmoid activation function used to constrain the mask values between 0 and 1. The deformable convolution operation is then formulated as Equation 5, where $Y(\;p_{0})$ represents the output feature at location $p_{0}$, *X* denotes the input feature map, $w_{k}$ represents the convolution kernel weights, $p_{k}$ indicates the regular sampling locations, $\Delta p_{k}$ represents the learned offsets, and $m_{k}$ denotes the modulation coefficient obtained from the learned mask.

$$\Delta P=Conv_{offset}(X)$$ (3)

$$M=\sigma (Conv_{mask}(X))$$ (4)

$$Y(\;p_{0})=\overset{K}{\underset{k=1}{\sum }}w_{k}\cdot X(\;p_{0}+p_{k}+\Delta p_{k})\cdot m_{k}$$ (5)

For maxillary sinus segmentation, DCNv2 provides an adaptive sampling capability that enables the network to better follow the anatomical variations of the left and right maxillary sinuses (22). This is particularly beneficial for CT-based segmentation, where sinus boundaries may vary between subjects and may be affected by pathological changes. By adjusting the receptive field according to the input features, DCNv2 improves boundary localisation and enhances the extraction of detailed anatomical information. In the proposed architecture, DCNv2 modules have been embedded in certain stages of the YOLO11n-Seg backbone network to replace the convolution operation. Such embedding allows feature adaptation while retaining the lightness property of the model.

### 3.3 Evaluation metrics

The performance of the proposed framework was evaluated using multiple metrics to assess segmentation accuracy, localisation capability, and computational efficiency (23). Since the proposed YOLO11n-Seg-based framework performs both object detection and pixel-level segmentation, the evaluation includes detection metrics (Precision and Recall), segmentation metrics (Mask mAP50 and Mask mAP50-95), and computational efficiency indicators (Parameters, GFLOPs, and Inference Time). In maxillary sinus segmentation using head CT scans, these metrics assess the capability of the model in the accurate identification of the left and right maxillary sinuses and produce an exact segmentation mask while retaining computational efficiency (24).

Precision is the ratio of true positive predictions to the total number of predictions made by the model, reflecting its efficiency in minimizing errors of positive predictions and avoiding misclassification of the nearby anatomical regions as the target regions, as depicted in Equation 6, where TP refers to the number of true positive predictions, and FP refers to the number of false positive predictions. Recall reflects the efficiency of the model in predicting all target regions available in the CT image scans. The higher the value of the recall, the fewer target regions are missed during the prediction process as depicted in Equation 7 below, where FN refers to the number of false negative predictions.

The segmentation performance was evaluated using Mask mean Average Precision at an Intersection over Union threshold of 0.5 (Mask mAP50). The measure assesses the amount of agreement between the predicted masks and the ground truth annotations. IoU is calculated based on the formula given in Equation 8. $A_{\;pred}$ refers to the area of the segmented masks predicted, while $A_{gt}$ stands for the area of the ground truth masks. Mask mAP50 is the measure of the average precision calculated at an IoU value of 0.5 and formulated in Equation 9, where AP represents the Average Precision obtained from the precision-recall curve. Alongside mAP50, a more rigorous metric called Mask mAP50-95. The Mask mAP50-95 metric determines the mean average precision based on several IoUs between 0.50 and 0.95 in increments of 0.05, as depicted in Equation 10. In contrast to the IoU metric that determines IoU at a point as in the mAP50, the Mask mAP50-95 offers a holistic approach to evaluate mask localization.

$$Precision(P)=\frac{TP}{TP+FP}$$ (6)

$$Recall(R)=\frac{TP}{TP+FN}$$ (7)

$$IoU=\frac{A_{\;pred}\cap A_{gt}}{A_{\;pred}\cup A_{gt}}$$ (8)

$$mAP_{50}=AP(IoU=0.5)$$ (9)

$$mAP_{50\text{-}95}=\frac{1}{10}\overset{9}{\underset{i=0}{\sum }}AP(IoU=0.50+0.05i)$$ (10)

In contrast to the mAP50 that assesses the performance of segmentation at one IoU threshold level, the metric mAP50-95 gives a more accurate evaluation of the mask localisation and boundaries precision on various levels of overlap. It is especially important in the case of maxillary sinus segmentation, where accurate definition of the anatomical boundaries is necessary to discriminate the sinus areas from the other surrounding structures on CT images. Besides the assessment of the segmentation performance, the computational efficiency of the suggested model was assessed by means of the following parameters: Parameters, GFLOPs, and Inference Time. Parameter is the measure of the complexity of the model and memory consumption, whereas GFLOPs are related to the computational costs during the process of inference. The inference time is the measure of the process speed for producing the detection and segmentation results from CT images. These indicators provide an assessment of the balance between segmentation accuracy and computational efficiency, which is important for developing a reliable and practical framework for maxillary sinus analysis.

## 4 Results

### 4.1 Experimental setup

All experiments were conducted on a dataset of 130 nasal cavity CT volumetric studies. Each 3-D volume was decomposed into individual 2-D axial slices, and only slices containing at least one of the two target structures, the right and/or left maxillary sinus, were retained, yielding a final dataset of 3,620 2-D image-mask pairs. The dataset was partitioned at the patient level, prior to slice extraction, into training (104 patients, 2,883 images), validation (13 patients, 355 images), and test (13 patients, 382 images) subsets, ensuring that slices from a given patient were confined exclusively to a single subset.

Training was performed on an NVIDIA GeForce RTX 4060 Ti (8 GB VRAM) running Python 3.10, PyTorch 2.5.1, and Ultralytics 8.4.24. All models, including every baseline and the proposed model, were trained for 50 epochs using an identical batch size of 16, with no model receiving a different batch size. The AdamW optimiser was applied automatically by the Ultralytics framework, with an initial learning rate of 0.01.

### 4.2 Quantitative results

#### 4.2.1 Bounding-box detection

Table 2 reports bounding-box detection performance on the held-out test set (382 slices from 13 patients) for all four models. The proposed model achieves the highest scores across all four COCO metrics, recording a Precision of 96.33%, Recall of 88.44%, mAP50 of 91.00%, and mAP50-95 of 75.83%.

**Table 2:** Bounding-box detection performance on the test set (382 slices, 13 patients).

Model	Precision (%)	Recall (%)	mAP50 (%)	mAP50-95 (%)
Proposed model	**96.33**	**88.44**	**91.00**	**75.83**
YOLO26n-Seg	94.41	85.77	88.65	73.83
YOLO11n-Seg	93.05	86.30	88.47	73.69
YOLOv8n-Seg	93.17	85.24	86.90	73.19

Bold green rows indicate the best result per column. ↑ higher is better.

Relative to the strongest baseline, YOLO26n-Seg, the proposed model improves box mAP50 by +2.35 pp and box mAP50-95 by +2.00 pp. Against the weakest baseline, YOLOv8n-Seg, improvements reach +4.10 pp in mAP50 and +2.64 pp in mAP50-95. YOLO26n-Seg and YOLO11n-Seg occupy closely matched intermediate positions, with YOLO26n-Seg achieving marginally higher Precision and mAP50 than YOLO11n-Seg, while YOLO11n-Seg attains a slightly higher Recall (86.30% vs. 85.77%); across all three baselines, the proposed model maintains a consistent margin on every reported metric.

#### 4.2.2 Instance-segmentation (mask) performance

Table 3 presents instance-segmentation metrics for the same test set (382 slices, 13 patients). The proposed model attains a mask mAP50 of 93.07% and mask mAP50-95 of 74.49%, the highest values among all compared architectures. Compared with YOLO11n-Seg, the strongest baseline on this metric, the improvements are +2.04 pp and +2.35 pp in mAP50 and mAP50-95, respectively. The widest margins observed are +4.60 pp in mAP50 relative to YOLO26n-Seg (the weakest baseline on this metric) and +3.30 pp in mAP50-95 relative to YOLOv8n-Seg (the weakest baseline on that metric).

**Table 3:** Instance-segmentation (mask) performance on the test set (382 slices, 13 patients).

Model	Precision (%)	Recall (%)	mAP50 (%)	mAP50-95 (%)
Proposed Model	**97.32**	**89.10**	**93.07**	**74.49**
YOLO11n-Seg	94.22	86.59	91.03	72.14
YOLOv8n-Seg	94.06	85.60	89.73	71.19
YOLO26n-Seg	92.87	85.94	88.47	72.07

Metrics follow the COCO evaluation protocol. Bold values indicate the best result per column.

It is worth noting that mask mAP50 exceeds box mAP50 for the proposed model and for two of the three baselines (YOLO11n-Seg: +2.56 pp; YOLOv8n-Seg: +2.83 pp), consistent with a general property of IoU-based evaluation: for a non-convex target region, the circumscribing bounding box contains background area not belonging to the object, making box IoU more sensitive to localization and scale errors than mask IoU, which is computed directly against the object's segmented footprint. However, this pattern does not hold universally: for YOLO26n-Seg, mask mAP50 (88.47%) is marginally lower than its box mAP50 (88.65%), indicating that the geometric relationship between box and mask IoU is not guaranteed to favour masks in every case and may depend on model-specific prediction behaviour rather than being a fixed property of the anatomical target alone. We therefore present the box-mask IoU relationship as a plausible, general geometric tendency rather than a universal or anatomically demonstrated cause, since a direct causal analysis relating shape irregularity to this gap was not performed in the present study. Among the models for which mask mAP50 does exceed box mAP50, the largest gap is observed for YOLOv8n-Seg (+2.83 pp) rather than the proposed model (+2.07 pp), further indicating that the magnitude of this gap is not systematically tied to the architectural modifications introduced in the proposed model.

### 4.3 Computational complexity

Table 4 summarises the architectural complexity and per-slice (2-D) inference latency of each model, measured on the same NVIDIA RTX 4060 Ti (8 GB VRAM) GPU used for training; no CPU inference measurements were performed. Mask mAP50 values reflect the updated 382-slice test set.

**Table 4:** Model complexity and inference speed comparison.

Model	Parameters	GFLOPs	Inference (per slice, GPU)	Mask mAP50 (%)
Proposed model	4.91M	15.1	27.2 ms	93.07
YOLO11n-Seg	2.83M	9.6	20.6 ms	91.03
YOLOv8n-Seg	3.26M	11.3	26.2 ms	89.73
YOLO26n-Seg	2.69M	9.0	14.2 ms	88.47

The proposed model is approximately 1.83× larger in parameter count and 1.68× more computationally demanding (GFLOPs) than the lightest baseline (YOLO26n-Seg). As discussed in Section 5.2, this substantial increase in capacity means the proposed model is no longer directly comparable to the “n”-variant baselines purely as a same-scale lightweight architecture, and part of the reported accuracy improvement may be attributable to this added capacity rather than exclusively to the CoordAtt/DCNv2 mechanisms; a parameter- or compute-matched baseline would be required to fully disentangle these factors.

### 4.4 Qualitative results

Figures 5, 6 present a qualitative comparison of bounding-box detection and instance segmentation outputs, respectively, for a representative axial CT slice across all four evaluated models.

**Figure 5:**
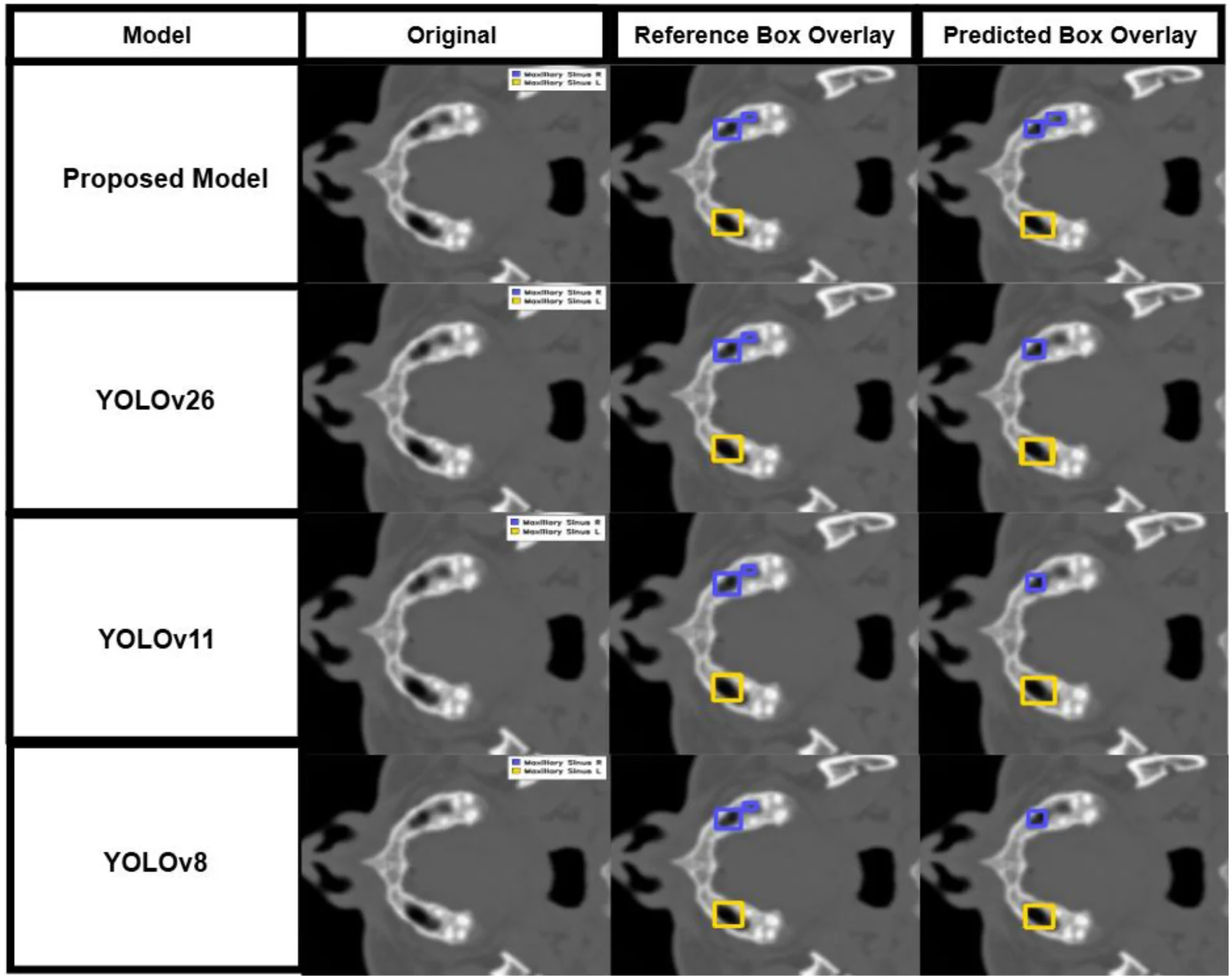
Qualitative comparison of bounding-box detection results for a representative axial CT slice. Blue and yellow boxes indicate the right and left maxillary sinuses, with the proposed model showing the closest match to the ground truth.

**Figure 6:**
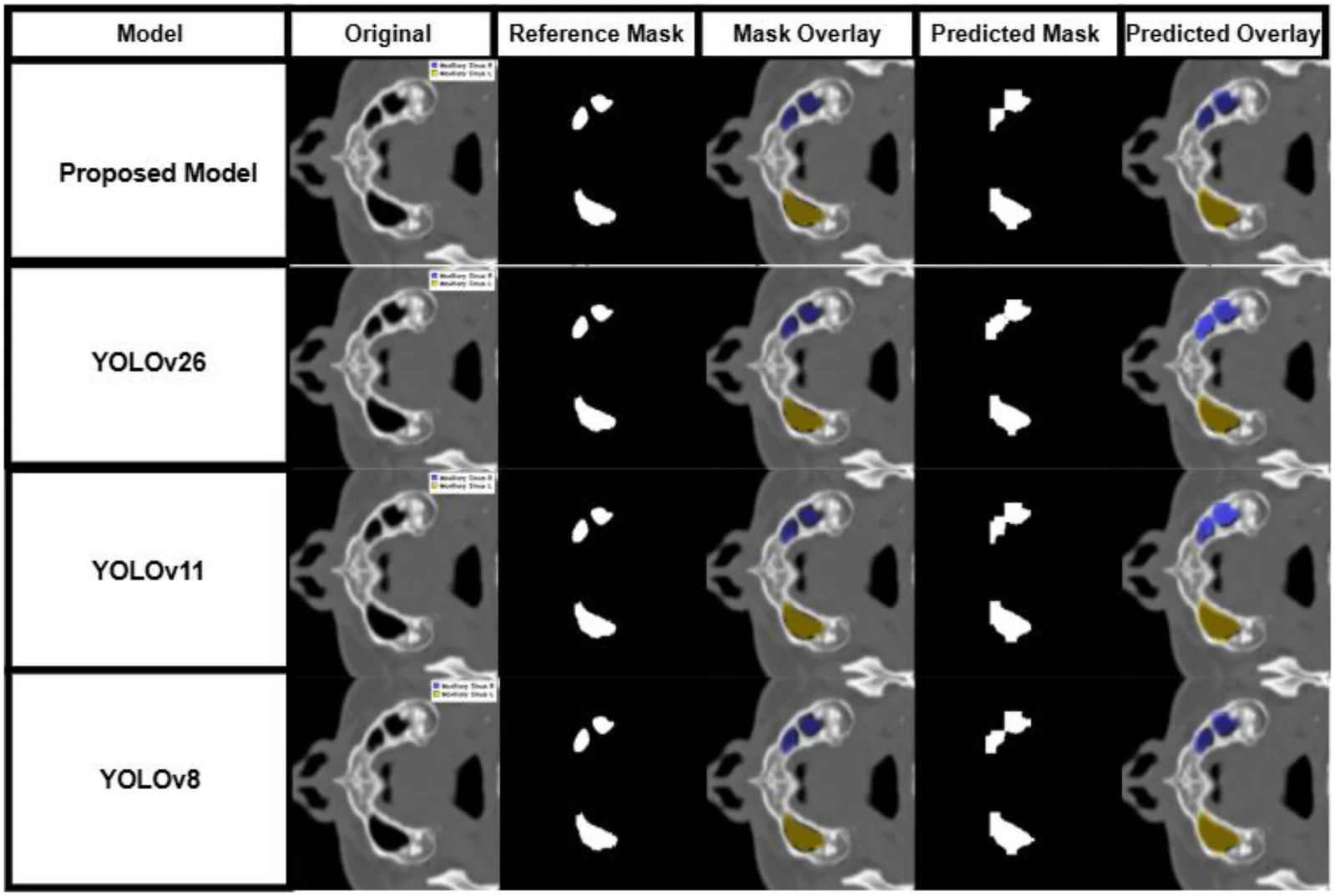
A qualitative comparison of segmentation outputs shows that the proposed model yields the most accurate bilateral maxillary sinus boundaries among all baseline architectures.

Figure 5 demonstrates the bounding box detection performance with the original CT scan along with the reference box overlay (ground truth) and the predicted box overlay from each of the models. The developed model is capable of detecting bounding boxes that are well matched with the ground truth localization for the right maxillary sinus (blue) and left maxillary sinus (yellow). Meanwhile, the YOLOv8 model shows significant localization inaccuracies in terms of both dimensions and positions of the detected boxes. YOLO11 and YOLO26 show intermediate localization performance compared to the developed model.

Figure 6 depicts the corresponding results of instance segmentation, with the reference mask, mask overlay, predicted mask, and predicted overlay displayed for each model. The output from the proposed model is characterized by segmentation masks, which fit the contour of the maxillary sinuses in a very close manner without any significant difference. On the other hand, the baseline models deteriorate progressively in terms of mask accuracy, where YOLO11 and YOLO26 create fairly accurate masks with small deviations at the boundaries, while YOLOv8 creates masks with significant under-segmentation of the right maxillary sinus.

Figure 7 presents a multi-case qualitative comparison across four test-set cases, rather than a single representative slice, spanning typical bilateral sinus involvement (Cases 1, 4), a smaller single-region case (Case 2), and a more anatomically challenging case with smaller, irregular boundaries (Case 3). For each case and model, we report true-positive/false-positive/false-negative (TP/FP/FN) difference maps directly overlaid on the CT slice green for true positives, red for false positives, blue for false negatives rather than simple ground-truth/prediction overlays, to more clearly localise segmentation errors.

**Figure 7:**
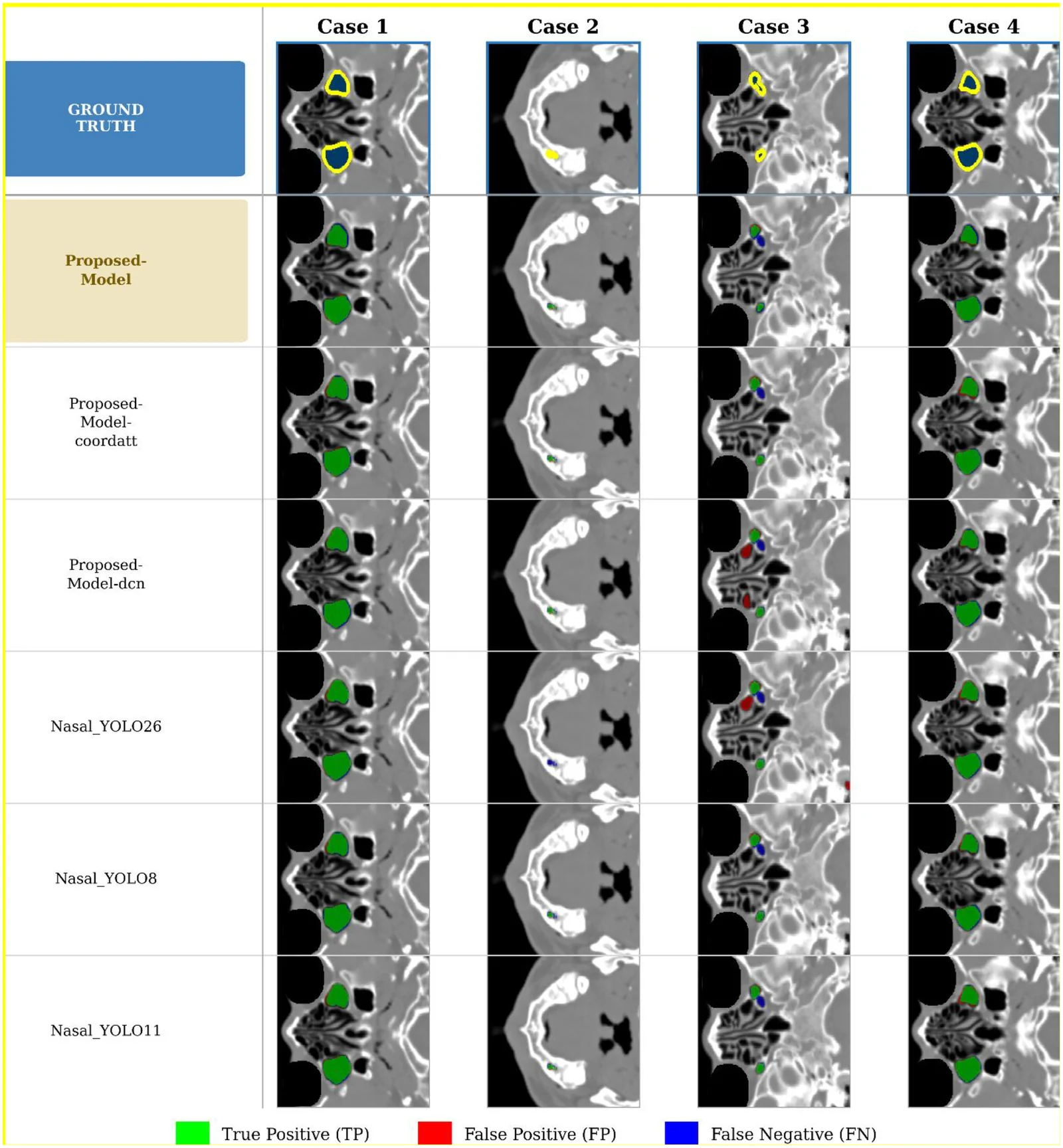
True-positive (green)/false-positive (red)/false-negative (blue) difference maps across four test-set cases, for the proposed model, its two ablation variants, and the three YOLO-family baselines, relative to ground truth (top row).

Across Cases 1, 2, and 4, all models achieve near-complete true-positive coverage with minimal errors. Case 3, the more challenging example, differentiates performance more clearly: the proposed model shows only minor false negatives and no false positives, while Nasal_YOLO26 shows both larger false negatives and a false positive not seen in the proposed model.

### 4.5 Ablation study

To isolate the individual and combined contributions of the two architectural modifications integrated into the proposed model Coordinate Attention (CoordAtt) and Deformable Convolutional Networks v2 (DCNv2) a component-wise ablation study was conducted. Four configurations were evaluated on the same held-out test set (382 slices from 13 patients): the YOLO11n-Seg baseline, YOLO11n-Seg with CoordAtt only, YOLO11n-Seg with DCNv2 only, and the complete proposed model (YOLO11n-Seg + CoordAtt + DCNv2). Table 5 reports bounding-box and instance-segmentation performance for each configuration.

**Table 5:** Component-wise ablation study on the test set (382 slices, 13 patients).

Configuration	Box P (%)	Box R (%)	Box mAP50 (%)	Box mAP50-95 (%)	Mask P (%)	Mask R (%)	Mask mAP50 (%)	Mask mAP50-95 (%)
YOLO11n-Seg (baseline)	93.05	86.30	88.47	73.69	94.22	86.59	91.03	72.14
+CoordAtt	91.52	84.53	87.24	72.25	94.50	85.23	90.51	72.22
+DCNv2	93.68	86.57	88.12	73.44	94.15	86.39	89.80	72.73
+CoordAtt + DCNv2 (Proposed)	**96.33**	**88.44**	**91.00**	**75.83**	**97.32**	**89.10**	**93.07**	**74.49**

Bold values indicate the best result per column. ↑ higher is better.

Individually, neither modification consistently improved upon the baseline: CoordAtt alone reduced box precision (93.05% → 91.52%) and box mAP50 (88.47% → 87.24%) while yielding only a marginal mask precision gain, and DCNv2 alone produced small, mixed changes across box and mask metrics without a clear overall improvement. Only the combination of CoordAtt and DCNv2 produced a consistent and substantial improvement across all eight detection and segmentation metrics, most notably raising mask mAP50-95 from 72.14% at baseline to 74.49% for the full model. This pattern indicates that the two modules are complementary rather than independently sufficient: CoordAtt's positional attention appears to compensate for the spatial feature dispersion introduced by DCNv2's learned sampling offsets, while DCNv2's adaptive receptive field enables CoordAtt to focus on anatomically relevant, irregularly shaped sinus boundaries.

### 4.6 Statistical reliability and uncertainty analysis

Given the limited size of the test cohort (13 patients, 382 slices), single-point performance estimates alone are insufficient to establish the robustness of the reported improvements. To address this, 95% confidence intervals were computed for all principal metrics using patient-level bootstrap resampling: patients, rather than individual slices, were resampled with replacement, and metrics were re-aggregated over each resampled cohort to construct the bootstrap distribution. This procedure preserves the correlation structure among slices originating from the same patient and avoids the artificially narrow confidence intervals that would result from treating slices as independent samples. Confidence intervals were computed both at the slice level (2-D Dice, IoU, HD95, and ASSD; Table 6) and at the patient level (volumetric Dice3D, HD95_3D, and NSD3D, aggregated per patient; Table 7), for both target classes. Pairwise comparisons between the proposed model and each baseline/ablation configuration were additionally assessed using the paired Wilcoxon signed-rank test, which is appropriate given the paired and non-normally distributed nature of per-case metric differences. The single *p*-value reported per row in Tables 6, 7 corresponds to the Dice metric only; the remaining metrics (IoU, HD95, ASSD, NSD3D) are reported descriptively and are not individually accompanied by a significance test in the current version of this table.

**Table 6:** 2-D (slice-level) segmentation metrics with 95% confidence intervals (patient-level bootstrap, *n* = 13 patients, 382 slices) and paired Wilcoxon signed-rank test *p*-values vs. the proposed model.

Configuration	Dice (95% CI)	IoU (95% CI)	HD95 mm (95% CI)	ASSD mm (95% CI)	*p* vs. proposed dice
(a) Class 1—right maxillary sinus					
YOLO11n-Seg (baseline)	0.602 [0.577, 0.626]	0.484 [0.457, 0.511]	5.12 [4.82, 5.41]	2.58 [2.44, 2.73]	<0.001
+CoordAtt	0.606 [0.580, 0.632]	0.484 [0.458, 0.512]	5.12 [4.82, 5.43]	2.58 [2.42, 2.74]	<0.001
+DCNv2	0.606 [0.581, 0.632]	0.485 [0.458, 0.510]	5.08 [4.79, 5.39]	2.54 [2.39, 2.68]	<0.001
Proposed (full)	**0.623 [0.597, 0.649]**	**0.503 [0.476, 0.530]**	**4.94 [4.66, 5.22]**	**2.48 [2.33, 2.62]**	–
(b) Class 2—left maxillary sinus					
YOLO11n-Seg (baseline)	0.605 [0.578, 0.631]	0.494 [0.467, 0.520]	4.71 [4.37, 5.10]	2.45 [2.30, 2.62]	<0.001
+CoordAtt	0.615 [0.589, 0.641]	0.503 [0.475, 0.529]	4.63 [4.32, 4.96]	2.40 [2.25, 2.56]	<0.001
+DCNv2	0.615 [0.588, 0.639]	0.501 [0.475, 0.530]	4.61 [4.30, 4.92]	2.43 [2.27, 2.58]	<0.001
Proposed (full)	**0.635 [0.608, 0.661]**	**0.519 [0.491, 0.546]**	**4.50 [4.20, 4.80]**	**2.36 [2.21, 2.51]**	–

Bold values indicate the best result per column.

**Table 7:** Volumetric (patient-level 3-D) segmentation metrics with 95% confidence intervals (patient-level bootstrap, *n* = 13 patients) and paired Wilcoxon signed-rank test *p*-values vs. the proposed model.

Configuration	Dice3D (95% CI)	HD95_3D (mm) (95% CI)	NSD3D (95% CI)	*p* vs. proposed dice
(a) Class 1—right maxillary sinus				
YOLO11n-Seg (baseline)	0.598 [0.515, 0.684]	3.67 [2.65, 4.68]	0.660 [0.577, 0.745]	<0.001
+CoordAtt	0.596 [0.510, 0.686]	3.69 [2.63, 4.74]	0.666 [0.580, 0.755]	<0.001
+DCNv2	0.589 [0.505, 0.677]	3.75 [2.66, 4.85]	0.656 [0.572, 0.742]	<0.001
Proposed (full)	**0.616 [0.527, 0.707]**	**3.59 [2.54, 4.63]**	**0.683 [0.596, 0.774]**	–
(b) Class 2—left maxillary sinus				
YOLO11n-Seg (baseline)	0.606 [0.516, 0.698]	3.46 [2.33, 4.64]	0.670 [0.581, 0.758]	<0.001
+CoordAtt	0.609 [0.517, 0.703]	3.47 [2.30, 4.69]	0.678 [0.587, 0.768]	<0.001
+DCNv2	0.601 [0.508, 0.696]	3.56 [2.33, 4.85]	0.661 [0.571, 0.753]	<0.001
Proposed (full)	**0.627 [0.531, 0.725]**	**3.40 [2.23, 4.63]**	**0.693 [0.599, 0.788]**	**–**

Bold values indicate the best result per column.

Across both target classes, Class 1 (Right Maxillary Sinus) and Class 2 (Left Maxillary Sinus), the proposed full model achieved the highest Dice3D and NSD3D values and the lowest HD95_3_D values among the evaluated configurations. For Class 1, Dice3D increased from 0.598 for the YOLO11n-Seg baseline to 0.616 for the proposed model, while for Class 2 it increased from 0.606 to 0.627. According to Table 7, all six paired comparisons between the proposed model and the baseline or single-component ablation models showed *p* < 0.001 for Dice3D. Nevertheless, the 95% confidence intervals overlap between the proposed model and the comparator models, which is consistent with the limited sample size of 13 independent patients. Therefore, the observed improvements should be interpreted cautiously and as modest but consistent differences in patient-level performance.

To complement the class-specific analysis, Table 8 reports the per-patient average of the right and left maxillary sinus metrics for each patient. This aggregated analysis provides an overall patient-level view of performance across both target sinuses. Based on the values reported in Table 8, the mean Dice3D of the Full model across the 13 patients is approximately 0.6215, with values ranging from 0.4014 for P130 to 0.9029 for P071. This overall mean lies between the class-specific Dice3D estimates reported for Class 1 (0.616) and Class 2 (0.627), supporting consistency between the aggregated patient-level results and the separate class-specific analyses.

**Table 8:** Per-patient averaged Dice3D, HD95_3_D, and NSD3D across both maxillary sinuses for the baseline and ablation models.

Patient ID	Dice3D				HD95_3_D (mm)				NSD3D			
	Base	+CA	+DC	Full	Base	+CA	+DC	Full	Base	+CA	+DC	Full
P007	0.7924	0.7864	0.7761	0.8023	1.526	1.879	1.702	1.516	0.8498	0.8493	0.8372	0.8649
P012	0.7454	0.7489	0.7323	0.7640	1.798	1.742	1.803	1.687	0.8175	0.8276	0.8074	0.8417
P014	0.6134	0.6149	0.6103	0.6279	3.602	3.561	3.742	3.555	0.6844	0.6930	0.6814	0.7045
P018	0.4397	0.3954	0.4266	0.4513	4.724	5.041	4.955	4.649	0.5031	0.4669	0.4946	0.5213
P029	0.5857	0.5726	0.5746	0.5827	3.827	3.862	3.983	3.781	0.6527	0.6477	0.6487	0.6577
P032	0.4583	0.4558	0.4472	0.4684	5.373	5.403	5.524	5.307	0.5193	0.5233	0.5122	0.5349
P070	0.3959	0.3979	0.3893	0.4064	7.278	7.354	7.610	7.257	0.4558	0.4628	0.4518	0.4709
P071	0.8426	0.8552	0.8623	0.9029	0.695	0.675	0.700	0.655	0.8889	0.9121	0.9206	0.9595
P087	0.5555	0.5555	0.5424	0.5696	3.561	3.661	3.369	3.309	0.6507	0.6598	0.6447	0.6719
P095	0.6350	0.6582	0.6461	0.6763	3.872	3.822	3.943	3.741	0.6703	0.7006	0.6829	0.7147
P115	0.8562	0.8587	0.8325	0.8804	1.209	0.675	0.695	0.705	0.9140	0.9241	0.8919	0.9425
P124	0.5424	0.5374	0.5212	0.5455	4.512	4.764	4.824	4.774	0.6109	0.6134	0.5943	0.6190
P130	0.3923	0.3964	0.3802	0.4014	5.726	5.631	5.959	5.675	0.4513	0.4598	0.4392	0.4613

The per-patient values in Table 8 also show a generally consistent advantage of the Full model across the three segmentation metrics, although the magnitude of improvement varies between patients. These aggregated data provide a useful overall view of patient-level performance but should be considered complementary to, rather than a replacement for, the class-specific statistical analyses in Table 7. Because the cohort contains only 13 independent patients and the absolute Dice3D differences between configurations are relatively small, typically on the order of 0.01–0.02, the results should be interpreted as consistent but modest improvements.

## 5 Discussion

### 5.1 Interpretation of quantitative findings

The consistent superiority of the proposed model indicates that the combination of Coordinate Attention (CoordAtt) and Deformable Convolutional Networks v2 (DCNv2) improves maxillary sinus segmentation. As detailed in the ablation study, this improvement is not attributable to either component in isolation: CoordAtt alone did not consistently improve precision box precision decreased relative to the baseline, while mask precision showed only a marginal gain and DCNv2 alone produced only a small and inconsistent change in mAP50-95. Only the joint combination of both components yielded the substantial, consistent gains reported below, indicating a synergistic interaction between the two mechanisms rather than an additive or separable contribution from each.

The most pronounced gains appear at the stricter mAP50-95 threshold. This metric penalises predictions that achieve high overlap at IoU = 0.50 but fail to maintain that overlap as the threshold is tightened toward 0.95, which demands near-pixel-perfect boundary alignment. The proposed model improves mask mAP50-95 by +2.35 pp over the YOLO11n-Seg baseline (74.49% vs. 72.14%) and box mAP50-95 by +2.14 pp (75.83% vs. 73.69%). The maxillary sinus is a structurally challenging target: its thin walls, concave recesses, and variable inter-patient morphology create boundaries that do not align well with axis-aligned rectangular receptive fields. Deformable convolutions are, in principle, well suited to this setting, as they learn geometry-dependent sampling offsets during training; however, as the ablation results show, this benefit was only realised when DCNv2 was combined with CoordAtt, and we therefore present this as a plausible contributing mechanism rather than an isolated, experimentally demonstrated cause.

### 5.2 Limitations

The 130-patient cohort and 13-patient test set are small; we address this with patient-level bootstrap confidence intervals and significance testing, though expanding the patient cohort remains important future work. Each model was trained once with a single seed, so training-stochasticity variability was not quantified. The proposed model's higher parameter count and GFLOPs relative to the baselines were not fully disentangled from the CoordAtt/DCNv2 mechanisms, as a compute-matched baseline was not trained. Comparison against dedicated 3-D volumetric architectures (e.g., nnU-Net) on the same split has not yet been performed. Ground-truth annotations lack a prospective multi-rater agreement assessment. All data come from a single institution and scanner protocol, so external multi-centre validation is required before any clinical deployment claim. Finally, all inference measurements were GPU-based (RTX 4060 Ti); no CPU-only benchmarking was performed.

## 6 Conclusion and future work

This study proposed a moderate-capacity instance segmentation framework for automatic bilateral maxillary sinus delineation from CT images, extending YOLO11n-Seg with Coordinate Attention (CoordAtt) and Deformable Convolutional Networks v2 (DCNv2) to enhance boundary precision in the anatomically complex bilateral maxillary sinus. Evaluated on the NasalSeg dataset (382 test slices from 13 patients) against three YOLO-family baselines under a unified experimental configuration, the proposed model achieved a mask mAP50 of 93.07% and mask mAP50-95 of 74.49%, outperforming the strongest baseline (YOLO11n-Seg: mask mAP50 91.03%, mAP50-95 72.14%) as well as all other compared architectures. A component-wise ablation study confirmed that this improvement arises from the joint use of CoordAtt and DCNv2 rather than from either mechanism alone, and patient-level bootstrap confidence intervals with paired significance testing confirmed the statistical robustness of the observed gains.

Several directions are planned to extend this work. First, a parameter- or compute-matched baseline will be trained to disentangle the contribution of CoordAtt and DCNv2 from the effect of increased model capacity, and a dedicated 3-D volumetric baseline (e.g., nnU-Net) will be trained and evaluated on the identical patient-level dataset split to enable direct benchmarking against native 3-D approaches. Second, the framework will be extended to multi-class segmentation covering all five NasalSeg anatomical structures to broaden its clinical utility. Third, knowledge-distillation strategies will be explored to reduce the computational footprint for deployment on resource-constrained clinical hardware, and CPU-only inference benchmarking will be conducted to substantiate deployability claims on clinical workstations without dedicated GPU acceleration. Fourth, training will be repeated across multiple random seeds to quantify training-stochasticity variability in addition to the patient-level sampling variability already reported. Finally, external validation on multi-centre, multi-scanner cohorts and investigation of semi-supervised pretraining on larger unlabelled CT datasets will be pursued to improve generalisation and support clinical translation.

## Data Availability

The original contributions presented in the study are included in the article/Supplementary Material, further inquiries can be directed to the corresponding author. The code used to preprocess the NasalSeg dataset, implement the proposed model, and reproduce the training and evaluation procedures described in this study is publicly available at: https://github.com/alhatamlehs-art/coordatt_dcn_full. The underlying NasalSeg CT imaging data are not redistributed in this repository and remain available from their original public source (10); the repository provides the scripts required to preprocess these data into the format used in this study.

## References

[1] Fokkens WJ Lund VJ Hopkins C Hellings PW Kern R Reitsma European position paper on rhinosinusitis and nasal polyps 2020. Rhinology. (2020) 58(Suppl 29):1–464. 10.4193/Rhin20.401 32077450

[2] Hastan D Fokkens WJ Bachert C Newson RB Bislimovska J Bockelbrink A. Chronic rhinosinusitis in Europe - an underestimated disease: a GA2LEN study. Allergy. (2011) 66(9):1216–23. 10.1111/j.1398-9995.2011.02646.x 21605125

[3] DeConde AS Soler ZM. Chronic rhinosinusitis: epidemiology and burden of disease. Am J Rhinol Allergy. (2016) 30(2):134–9. 10.2500/ajra.2016.30.4297 26980394

[4] Harnsberger HR. Diagnostic imaging: head and neck (2004).

[5] Pulickal GG Navaratnam AV Nguyen T Dragan AD Dziedzic M Lingam RK. Imaging sinonasal disease with MRI: providing insight over and above CT. Eur J Radiol (2018) 102:157–68. 10.1016/j.ejrad.2018.02.033 29685531

[6] Bui NL Ong SH Foong KWC. Automatic segmentation of the nasal cavity and paranasal sinuses from cone-beam CT images. Int J Comput Assist Radiol Surg (2015) 10(8):1269–77. 10.1007/s11548-014-1134-5 25503593

[7] Darbari Kaul R Zhong W Liu S Azemi G Liang K Zou E. Development of an open-source algorithm for automated segmentation in clinician-led paranasal sinus radiologic research. Laryngoscope. (2025) 135(11):4125–33. 10.1002/lary.32292 40421828 PMC12645370

[8] Pani A Zedda L Mura DA Loddo A Di Ruberto C. 3D-NASE: a novel 3D CT nasal attention-based segmentation ensemble. J Imaging. (2025) 11(5):148. 10.3390/jimaging11050148 40423005 PMC12112669

[9] Wan S-Y Chen W-Y. Adaptive frequency-spatial dual-stream network (AFS-DSN) for nasal and paranasal sinus CT segmentation. IEEE Access. (2026) 14:68720–37. 10.1109/ACCESS.2026.3689347

[10] Zhang Y Wang J Pan T Jiang Q Ge J Guo X. Nasalseg: a dataset for automatic segmentation of nasal cavity and paranasal sinuses from 3D CT images. Sci Data. (2024) 11(1):1329. 10.1038/s41597-024-04176-1 39639065 PMC11621807

[11] Alam MZ Zain AM Hassan R Khattak AS. UHCT-Net: a unified hybrid CNN–transformer network for nasal CT segmentation and tumor analysis. IEEE Access. (2026) 14:46737–48. 10.1109/ACCESS.2026.3673705

[12] Möller H Krautschick L Graf R Atad M Busch C-J Beule AG. PARASIDE: an automatic paranasal sinus segmentation and structure analysis tool for magnetic resonance imaging. Comput Biol Med (2026) 204:111511. 10.1016/j.compbiomed.2026.111511 41619365

[13] Ullah N Algamdi SA Sadad T. Explainable hybrid CNN-transformer with self-supervised learning for structural analysis of paranasal sinus CT. Front Comput Neurosci (2026) 20:1778260. 10.3389/fncom.2026.1778260 42211246 PMC13214131

[14] Sun Y Guerrero-López A Arias-Londoño JD Godino-Llorente JI. Automatic semantic segmentation of the osseous structures of the paranasal sinuses. Comput Med Imaging Graph. (2025) 123:102541. 10.1016/j.compmedimag.2025.102541 40187116

[15] Hwang I-K Kang S-R Yang S Kim J-M Kim J-E Huh K-H. SinusC-Net for automatic classification of surgical plans for maxillary sinus augmentation using a 3D distance-guided network. Sci Rep (2023) 13(1):11653. 10.1038/s41598-023-38273-9 37468515 PMC10356765

[16] Altun O Özen DÇ Duman ŞB Dedeoğlu N Bayrakdar İŞ Eşer G. Automatic maxillary sinus segmentation and pathology classification on cone-beam computed tomographic images using deep learning. BMC Oral Health. (2024) 24(1):1208. 10.1186/s12903-024-04924-0 39390490 PMC11468140

[17] Ding Z Zhang Y Zhu C Zhang G Li X Jiang N. CAT-Unet: an enhanced U-net architecture with coordinate attention and skip-neighborhood attention transformer for medical image segmentation. Inf Sci. (2024) 670:120578. 10.1016/j.ins.2024.120578

[18] Chen S Qiu C Yang W Zhang Z. Multiresolution aggregation transformer UNet based on multiscale input and coordinate attention for medical image segmentation. Sensors. (2022) 22(10):3820. 10.3390/s22103820 35632229 PMC9145221

[19] Xu T Yuan Z. Convolution neural network with coordinate attention for the automatic detection of pulmonary tuberculosis images on chest x-rays. IEEE Access. (2022) 10:86710–7. 10.1109/ACCESS.2022.3199419

[20] Zhang L Li Y Qin S Men S Yang Z Huo L. DA-FRNet: a continuity-focused deformable attention network for retinal vessel segmentation in OCTA.

[21] Yi Z. Hybrid deformable and basic convolution network for fine retinal vessel segmentation. In: 2026 International Conference on Image, Signal Processing and Pattern Recognition (ISPP) (IEEE) (2026), 301–6

[22] Jian W Zhu L Zhang B He Q Yang G Jin H. Megavoltage CT enhancement for cervical cancer tomotherapy using a generative adversarial network with deformable convolution and self-attention. Med Phys (2026) 53(4):e70416. 10.1002/mp.70416 41968645

[23] Ali ML Zhang Z. The YOLO framework: a comprehensive review of evolution, applications, and benchmarks in object detection. Computers. (2024) 13(12):336. 10.3390/computers13120336

[24] Alanazi H. Optimizing medical image analysis: a performance evaluation of YOLO-based segmentation models. Int J Adv Comput Sci Appl. (2025) 16(4):1167–74. 10.14569/IJACSA.2025.01604111

[25] Bu X Zhang M Zhang Z Zhang Q. Computer-aided diagnoses for sore throat based on dynamic uncertain causality graph. Diagnostics. (2023) 13(7):1219. 10.3390/diagnostics13071219 37046437 PMC10093466

